# Recent advances in the genomic resources for sheep

**DOI:** 10.1007/s00335-023-10018-z

**Published:** 2023-09-26

**Authors:** Shernae A. Woolley, Mazdak Salavati, Emily L. Clark

**Affiliations:** 1 The Roslin Institute, University of Edinburgh, Easter Bush, Midlothian, EH25 9RG UK; 2 Scotland’s Rural College, Parkgate, Barony Campus, Dumfries, DG1 3NE UK

## Abstract

Sheep (*Ovis aries*) provide a vital source of protein and fibre to human populations. In coming decades, as the pressures associated with rapidly changing climates increase, breeding sheep sustainably as well as producing enough protein to feed a growing human population will pose a considerable challenge for sheep production across the globe. High quality reference genomes and other genomic resources can help to meet these challenges by: (1) informing breeding programmes by adding a priori information about the genome, (2) providing tools such as pangenomes for characterising and conserving global genetic diversity, and (3) improving our understanding of fundamental biology using the power of genomic information to link cell, tissue and whole animal scale knowledge. In this review we describe recent advances in the genomic resources available for sheep, discuss how these might help to meet future challenges for sheep production, and provide some insight into what the future might hold.

## Introduction

The domestic sheep (*Ovis aries*) is an important farmed animal species providing a source of protein and fibre to human populations across the globe. Sheep have excelled over the centuries in a range of production systems and environments (Mignon-Grasteau et al. 2005; Marshall et al. 2014; Alberto et al. 2018). Production systems differ across the globe, often with arable land, breed, environment, and key local and international markets playing a role in the type of production system used. The sheep industry in the United Kingdom, for example, is primarily based on sheep meat production, where the stratified system consists of three sectors: hill, upland and lowland, each utilising different breeds and production systems (Conington et al. 2001). The UK sheep sector currently largely uses traditional breeding practices, with a few exceptions, while in other countries such as Australia and New Zealand advanced genomics enabled breeding schemes have been widely implemented (Daetwyler et al. 2010; Brito et al. 2017a). Sheep production systems in place in countries that produce a large amount of sheep meat, including the UK, Australia and New Zealand rely on a relatively small number of popular breeds, to support large export markets. In contrast sheep production within low and middle income countries (LMICs) is orientated towards small-holder systems that make use of a diverse range of breeds that are adapted to harsh climatic and nutritional conditions (Marshall et al. 2019). In LMICs sheep production is vital to the livelihoods and nutritional needs of both individuals and communities, and often plays a multifaceted role within society (Marshall et al. 2019).

**Fig. 1:**
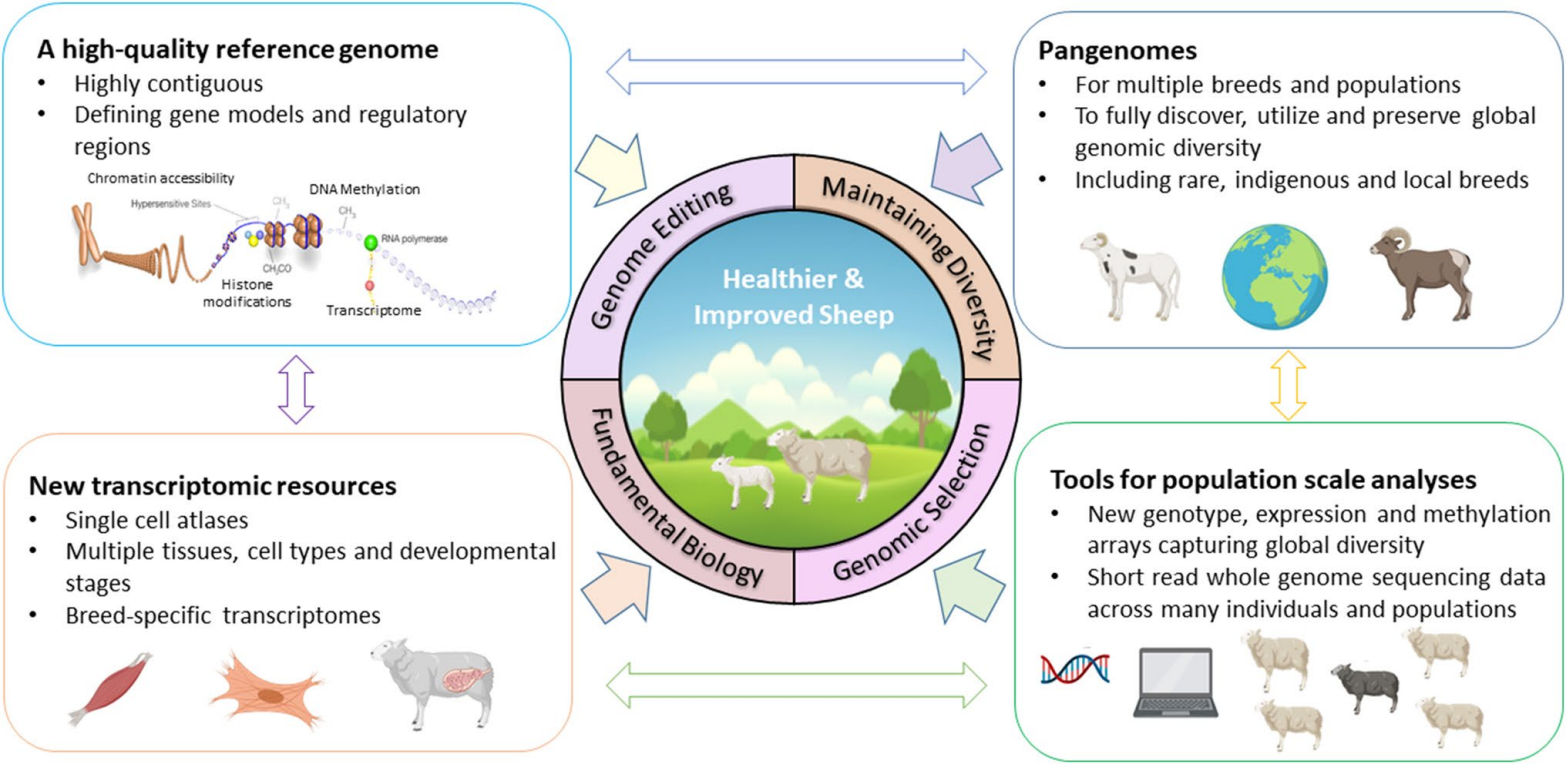
Schematic describing how new genomic resources for sheep will help to inform sheep breeding with the goal of providing healthier and improved animals, to meet growing pressures on food production, while maintaining genomic diversity (adapted from Clark et al. 2020). (Figure created using BioRender https://www.biorender.com/)

The future of sheep production, and its contributing role in global food production, will become more apparent in coming decades, due to predicted extremes of climate, and a growing human population that is expected to reach almost 9 billion by 2050 (McKenzie and Williams 2015). Any increase in global food production from sheep needs to be achieved with societal expectations around animal health and welfare in mind and should be guided through initiatives for responsible animal breeding such as Code EFABAR (EFFAB 2020). Sheep are also a source of greenhouse gases (Marino et al. 2016), and ambitious targets are being set to cut greenhouse gas emissions across the globe by 2030. Meeting these targets will require breeding strategies that reduce environmental impact (Mollenhorst and de Haas 2019). In addition, future breeding programmes will need to maintain genetic diversity for performance and resilience in the face of climatic extremes and other pressures (Dumont et al. 2020). In coming years breeding sheep sustainably using fewer resources, whilst flexibly meeting societal expectations, as well as producing enough protein to feed a growing human population, will pose a considerable challenge for sheep breeders and producers across the globe (Hayes et al. 2013). High quality reference genomes and other genomic tools and resources can help to meet these challenges (Clark et al. 2020). For example, they can: (1) inform breeding programmes including those enabled by genomic selection and genome editing (Georges et al. 2019), (2) provide tools for characterising and conserving genetic diversity (Talenti et al. 2022), and (3) improve our understanding of fundamental biology to link cell, tissue and whole animal scale knowledge (Giuffra and Tuggle 2019) (Fig. 1). Here we describe recent advances in the genomic resources available for sheep, discuss how these might help to meet future challenges for sheep production, and provide some insight into potential future opportunities.

## Towards a high quality highly contiguous reference genome for sheep

The genomic resources for sheep have gradually been improving in quality and resolution over the last twenty years in parallel with advances in sequencing technology. This is particularly evident when describing improvements in the quality and contiguity of the reference genome for sheep. The reference genome is the version of the sheep genome accepted by the sheep genomics community as a standard for comparison to sequence information generated in their own studies. A contiguous, high quality, well annotated and assembled reference genome for sheep is a hugely valuable research tool, providing a searchable map of the genome including the locations of expressed and regulatory regions. There have been several versions of the reference genome for sheep and each new version has kept pace with advancements in sequencing technology, starting with the ovine radiation hybrid panel (Cockett 2006). The first true version of a reference genome sequence for sheep (Ovis_aries_1.0; GCA_000005525.1) was a guided assembly using the bovine genome. It was generated from six female sheep of different breeds sequenced at 0.5 × coverage by 454 FLX (Dalrymple et al. 2007). Seven years later in 2014 the Texel reference genome Oar_v3.1 (GCA_000298735.1), assembled from two unrelated Texel sheep using Illumina short read sequencing at 150 × coverage, was released (Jiang et al. 2014). This assembly offered an improved contiguity (N50 contig length of approximately 40 Kb) and a genome length of 2.6 Gb (Jiang et al. 2014) (Table 1). The Oar_v3.1 genome assembly revealed segmental duplications within Texel sheep, along with a large run of homozygosity that contained the *MSTN* gene (Jiang et al. 2014). Previously a variant in the 3′ UTR region in the *MSTN* gene, that disrupted miRNA binding, had been shown to control the muscle hypertrophy (double muscling) phenotype in Texel sheep (Clop et al. 2006). The Oar_v3.1 reference genome provided a resource to interrogate the genomic regions associated with muscling in Texel sheep in more detail including the *MSTN* gene and the Texel muscling QTL (TM-QTL) on chromosome 18 (Macfarlane et al. 2014).

**Table 1:** Genome summary statistics for popular sheep reference genome sequence releases, and including goat for comparison, based on information from the National Centre for Biotechnology Information (NCBI) genome database

Genome assembly	Breed	Genome size (Mb)	Number of contigs	Contig N50 length	Contig L50 length
Ovis_aries_1.0 (GCA_000005525.1)	Mixed	2861	2,352,347	685	545,914
Oar_v3.1 (GCA_000298735.1)	Texel	2619	130,764	40,376	18,404
Oar_v4.0 (GCA_000298735.2)	Texel	2616	48,481	150,472	5008
Oar_rambouillet_v1.0 (GCA_002742125.1)	Rambouillet	2870	7486	2,572,683	313
ARS-UI_Ramb_v2.0 (GCA_016772045.1)	Rambouillet	2628	226	43,178,051	24
ARS1 (GCA_001704415.1)	Goat	2923	30,399	26,244,591	32

More recently, long read sequencing technologies capable of generating contiguous reads of greater than 10 Kb in length have provided a means to significantly improve the contiguity of a reference genome sequence (Pollard et al. 2018). A combination of Illumina^®^ GAII sequencing, Roche 454 sequencing and PacBio^®^ RSII technologies were used to gap fill Oar_v3.1 generating the more contiguous Texel Oar_v4.0 (GCA_000298735.2) genome (Table 1). Oar_v3.1 and Oar_v4.0 remained the gold standard reference genome sequences for sheep until 2020 when a new reference genome sequence was released that was generated using both Illumina^®^ HiSeq X short reads and PacBio^®^ RS II long read technology. This new reference genome assembly Oar_rambouillet_v1.0 (GCA_002742125.1) was built from the DNA of a single Rambouillet ewe Benz2616 (Liu et al. 2016). Oar_rambouillet_v1.0 had fewer contigs and a considerably greater contig N50 length than Oar_v3.1 and Oar_v4.0, replacing the Texel as the new reference genome for sheep (Table 1).

In 2022 a de novo assembly of the same Rambouillet ewe used to generate the Oar_rambouillet_v1.0 assembly was published, ARS-UI_Ramb_v2.0 (GCA_016772045.1) (Davenport et al. 2022). This new assembly was built using ∼50 × coverage Oxford Nanopore^®^ PromethION reads (N50 47 kb) and 75 × coverage Pacific Biosciences (PacBio) reads (N50 13 kb), with Hi-C data for scaffolding and Illumina short read data for final polishing (Davenport et al. 2022). The result was a 15-fold improvement in contiguity and increased accuracy over Oar_rambouillet_v1.0 (Table 1). The ARS-UI_Ramb_v2.0 genome is now the community adopted reference genome for sheep . It has provided the sheep genomics community with a very high quality reference genome assembled into fewer contigs than even the ARS1 goat genome (Table 1), which at the time of its release in 2017 was considered the gold standard of farmed animal genomes (Bickhart et al. 2017; Worley 2017).

## Annotation of regulatory regions in the reference genome by the Ovine FAANG project

High resolution annotation information, that accurately defines gene models and regulatory regions, adds basic functional genomic knowledge to the reference genome sequences for farmed animals increasing their power and utility as research tools (Georges et al. 2019; Giuffra and Tuggle 2019; Clark et al. 2020). The USDA NIFA funded Ovine FAANG project, led by the University of Idaho, provided the opportunity to annotate regulatory genomic regions in the new Rambouillet genome (Murdoch 2019). The Functional Annotation of Animal Genomes (FAANG) consortium is a concerted international effort to use molecular assays, developed during the Human ENCODE project (Birney et al. 2007), to annotate the majority of functional elements in the genomes of domesticated animals (Andersson et al. 2015; Giuffra and Tuggle 2019). By applying a set of core assays defined by the FAANG consortium, including five ChIP-Seq marks, ATAC-Seq, CAGE-Seq, RNA-Seq and methylation information, across a set of 56 tissues from Benz2616, the Ovine FAANG project developed a set of deep and robust expressed elements and regulatory features in the Rambouillet genome (Murdoch 2019). Some of these datasets are already available, via the FAANG Data Portal (https://data.faang.org/dataset?species=Ovis%20aries) (Harrison et al. 2021), including the CAGE dataset which provides a high resolution annotation of transcription start sites in the Oar_rambouillet_v1.0 genome (Salavati et al. 2020). RefSeq, and Ensembl, have also provided annotations of the coding regions for ARS-UI_Ramb_v2.0 (GCF_016772045.1) using the mRNA-Seq, CAGE and Iso-Seq data. Once the ATAC-Seq and ChIP-Seq data become available it will be possible for Ensembl to incorporate them into a regulatory build (Zerbino et al. 2015) annotating features of the genome that are involved in regulating gene expression . The Ovine FAANG project provides a valuable resource to facilitate a deeper understanding of how the regulatory regions of the genome control complex traits in sheep. It also provides a foundation for comparative analysis with other farmed animal species in which similar annotation datasets are available e.g. for cattle, chicken, goat and pig (Foissac et al. 2019; Goszczynski et al. 2021; Kern et al. 2021).

From the human literature we know that as many as 90% of variants underlying complex traits identified in Genome Wide Association Studies (GWAS) are located in non-coding regions of the genome (Tam et al. 2019). In addition to the efforts of the Ovine FAANG project in annotating the new Rambouillet reference genome sequence, there have been a small number of other studies to date that have characterised regulatory regions in the sheep genome. For example, Davenport et al. (2021) used histone modifications that distinguish active or repressed chromatin states, CTCF binding, and DNA methylation to characterize regulatory elements in liver, spleen, and cerebellum tissues from four yearling sheep to identify the regulatory regions of genes that play key roles in defining health and economically important traits. To evaluate the impact of selection and domestication on regulatory sequences Naval-Sanchez et al. (2018) used histone modification and gene expression data. Their analyses showed that selective sweeps were significantly enriched for protein coding genes, proximal regulatory elements of genes and genome features associated with active transcription. In addition, they were able to show that remodelling of gene expression is likely to have been one of the evolutionary forces driving phenotypic diversification in domestic sheep (Naval-Sanchez et al. 2018). Both studies demonstrate the value of regulatory annotation information in understanding the genomic processes driving gene expression and shaping the characteristics and genetic diversity of global sheep populations.

## Annotating expressed regions in the sheep genome, the sheep gene expression atlas and beyond

Advances in transcriptome sequencing technology and reductions in cost have also led to improvements in annotation of the expressed regions of the sheep genome over the last decade. Coding regions in the Oar_v3.1 reference genome (Jiang et al. 2014) were annotated by Ensembl with their ‘Genebuild’ pipeline (Aken et al. 2016) using RNA-sequencing data from more than 80 tissues collected from a Texel ewe, lamb and ram trio (http://useast.ensembl.org/Ovis_aries/Info/Annotation). When released the Oar_v3.1 annotation was one of the most comprehensive annotations of any of the farmed animal species and was widely used by the community until Oar_rambouillet_v1.0 was annotated by Ensembl in 2020 (http://www.ensembl.org/Ovis_aries_rambouillet/Info/Annotation). Over the last decade a vast amount of RNA-sequencing data for sheep has been generated, capturing global transcriptomic complexity across multiple tissues, cell types and developmental stages (Jiang et al. 2014; Clark et al. 2017). In 2017 a large-scale gene expression atlas (http://biogps.org/sheepatlas) was generated from tissues and cells collected from all of the major organ systems from adult Texel × Scottish Blackface sheep and from juvenile, neonatal and prenatal developmental stages (Clark et al. 2017). Of the 20,921 protein coding genes, that were annotated in the Oar v3.1 reference genome, 19,921 (92%) had detectable expression in at least one tissue in the sheep gene expression atlas dataset (Clark et al. 2017). Network-based cluster analysis, using the software package Graphia (Freeman et al. 2022), was used to describe the overall transcriptional signatures present in the sheep gene expression atlas and assign those signatures, where possible, to specific tissues or cell types.

The next frontier for the sheep transcriptome will be to fully resolve the tissue- and cell- type specific transcriptional signatures generated for the sheep atlas from bulk tissue samples, at a single cell resolution. Single-cell sequencing technologies enable the deconvolution of transcriptional and regulatory complexity in tissues comprised of many different cell types e.g. (Schaum et al. 2018). Atlases of gene expression generated using single cell sequencing technologies have already been created for pig (https://dreamapp.biomed.au.dk/pigatlas/) (Wang et al. 2022). Building similar single cell transcriptomic resources for sheep from multiple tissue types and developmental stages and adding regulatory information with single cell ATAC-seq, for example, would provide insights into cell composition, cell-to-cell interactions and the cellular heterogeneity of tissues. As datasets of this type are generated for more species of farmed animals sets of cell specific marker genes that are conserved across species will be revealed. Such markers could be applied as a proxy for a particular cell type e.g. (Herrera-Uribe et al. 2021) and may be useful as a costly but high value intermediate phenotype for complex trait prediction, providing a powerful tool for linking genotype to phenotype in sheep and other farmed animal species.

## The power of pangenomes: moving beyond a single reference genome sequence

Recent advances in long read sequencing technologies, and reductions in cost, have meant that in addition to a single very high quality highly annotated reference genome per farmed animal species it is now possible to generate chromosome level (relatively complete) genomes for many different breeds and populations. Many new chromosome level genomes including, for example, for Hu sheep (Li et al. 2021), Dorper (Qiao et al. 2022), and Tibetan sheep (Li et al. 2022) have recently been deposited in NCBI (Table 2). In addition, a pangenome for sheep has been generated that includes new long read assemblies for 13 different breeds (Li et al. 2023). Currently, NCBI reports that there are 55 genome assemblies for sheep (https://www.ncbi.nlm.nih.gov/data-hub/genome/?taxon=9940). Some of these are alternate-pseudohaplotypes, where two pseudohaplotype assemblies of the diploid genome have been generated, and each release of the reference genome sequences for the Rambouillet and Texel are also included in the database. In total, at the time of writing this review, there were 19 unique breeds of sheep that have chromosome level assemblies (Table 2), available in NCBI’s repository of genomes. These breeds represent 11 different countries (Table 2), and include the Suffolk, a British breed, that is a very popular terminal sire across the globe (https://www.suffolksheep.org/history/), and the Dorper a versatile composite that is used extensively for production in tropical regions (http://agtr.ilri.cgiar.org/dorper). Assembly statistics for the Rambouillet reference genome sequence (ARS-UI_Ramb_v2.0) are included in Table 2 to demonstrate that the majority of these new genome assemblies, generated using long read sequencing technologies, are close to reference quality in terms of contiguity.

**Table 2:** Chromosome level assemblies for breeds of sheep listed in NCBI, including basic assembly statistics and GenBank accessions, and with the reference genome ARS-UI_Ramb_v2.0 for comparison

Breed	Country	GenBank accession	Contig N50 (Mb)	No. of Contigs	Publication
Yunnan	China	GCA_022416785.1	71.9	1354	Li et al. (2023)
Chinese Merino	China	GCA_022432825.1	60	1773	Li et al. (2023)
Qaioke	China	GCA_022416685.1	75	1654	Li et al. (2023)
Hu	China	GCA_011170295.1	8.7	4131	Li et al. (2021)
Tibetan	Tibet	GCA_017524585.1	74.6	168	Li et al. (2022)
Kermani	Iran	GCA_022432835.1	80.3	1678	Li et al. (2023)
Kazak	Kazakhstan	GCA_022432845.1	73.4	1851	Li et al. (2023)
Ujimqin	Mongolia	GCA_022416755.1	75.7	1539	Li et al. (2023)
Waggir	Afghanistan	GCA_024222265.1	73.6	843	Li et al. (2023)
Texel	Netherlands	GCA_022416775.1	47.6	1838	Li et al. (2023)
Romney	UK	GCA_022538005.1	68.3	1553	Li et al. (2023)
Suffolk	UK	GCA_022416725.1	64.5	1520	Li et al. (2023)
Charollais	UK	GCA_022416745.1	65.1	1430	Li et al. (2023)
Polled Dorset	UK	GCA_022416915.1	92.4	1297	Li et al. (2023)
East Friesian	Germany	GCA_018804185.1	85.3	972	Qiao et al. (2022)
Romanov	Russia	GCA_024222175.1	31.8	1179	Li et al. (2023)
Romanov	Russia	GCA_022244705.1	62.3	499	–
Dorper	South Africa	GCA_019145175.1	73.3	142	Qiao et al. (2022)
White Dorper	South Africa	GCA_022416695.1	17.9	2133	Li et al. (2023)
White Dorper	South Africa	GCA_022244695.1	61.8	1178	–
Rambouillet (ARS-UI_Ramb_v2.0)	France	GCA_016772045.1	43.2	225	Davenport et al. (2022)

The number of breeds and populations with chromosome level genome assemblies will rise significantly as global pangenome efforts that aim to capture the global diversity of sheep breeds gather pace. The concept of a ‘pangenome’ is probably most simply defined as ‘any collection of genomic sequences to be analysed jointly or to be used as a reference’ (The Computational Pan-Genomics Consortium 2018). The USDA NIFA Ovine Pangenome Project, for example, plans to generate eight new haplotype-resolved assemblies from crosses of breeds selected for their divergent characteristics, using the trio-binning approach developed by Koren et al. 2018. For trio-binning usually an F1 cross of two disparate breeds of sheep, chosen to maximise heterozygosity, is generated. The genome assembly then relies on using short read Illumina data from the two parental genomes to first partition the long reads from the offspring into haplotype-specific sets. Each parental haplotype is then assembled independently, resulting in a complete diploid reconstruction, and effectively two new reference assemblies, one for each of the two parental breeds (Koren et al. 2018). This strategy has proved very successful in cattle (Koren et al. 2018; Rice et al. 2020) and has been used so far to produce the White Dorper × Romanov haplotype assemblies for sheep Oar_ARS-UKY_Romanov_v1.0 (GCA_022244705.1) and Oar_ARS-UKY_WhiteDorper_v1.0 (GCA_022244695.1) (Table 2).

These new chromosome level assemblies for sheep will improve our understanding of genome diversity and the drivers of breed-specific characteristics. As such global pangenome efforts should aim to capture the genomic diversity of global sheep populations. Understanding global genomic diversity provides a foundational resource for breed improvement and for the adaptation of sheep populations to changing environments and changing demands (FAO 2015). The United Kingdom’s native sheep breeds, for example, have become the mainstay of sheep production across the globe and as such capturing the genomic diversity represented by these breeds should be a priority (Bowles 2015; Romanov et al. 2021). This is particularly important in the context of breed conservation as many of the UK breeds, including for example the Norfolk Horn the ancestor of the Suffolk, are rare and declining in numbers (https://www.rbst.org.uk/norfolk-horn). Many European rare and indigenous breeds exhibit widespread heterozygote deficit due to declining diversity and are being lost due to introgression into large commercial populations (Lawson Handley et al. 2007). In LMICs where small-holder farmers rely on a wide diversity of breeds adapted to local conditions (Marshall et al. 2019), capturing the genomic diversity of indigenous African breeds is also important. For example, West and Central African indigenous breeds, such as the Cameroon sheep, represent a unique reservoir of genetic diversity and have followed the tracks of human migration across the globe contributing to the formation of Caribbean hair sheep breeds (Spangler et al. 2017; Wiener et al. 2022). The Cameroon sheep is also anecdotally thought to be trypanotolerant (Geerts et al. 2009). Genomic drivers of adaptation in local indigenous breeds to specific environmental challenges, including resistance or tolerance to specific diseases, need to be better understood (FAO 2015). Genomic information provided by global pangenome efforts for sheep should help to remedy this through comparative approaches, such as those described in Dutta et al. 2020 for water buffalo and cattle populations, to identify loci present in one breed, species or population that are missing in another.

Reference quality genome sequences representing the global diversity of sheep breeds also provide genomic resources that are relevant in a country or continent specific context. This is important because it can minimise reference mapping bias when working with short read whole genome sequencing data (Chen et al. 2021). For example, for a study investigating population genomics in sheep from the African continent using short read data, the Dorper (Qiao et al. 2022) a South African breed, would be a more appropriate reference assembly than the European Texel or Rambouillet. However, even when reference genome sequences for multiple different breeds are available the use of reference genome sequences that represent only a single individual, for understanding population diversity at the genomic level are still limited. There are two main reasons for this (described in Talenti et al. 2022); (i) because a single reference genome sequence represents one consensus haplotype of a single individual, and as such it would be expected that large sections of the diversity represented in the global pangenome for sheep will be missing, and (ii) reference mapping bias causes downstream analyses to be biased towards the alleles and haplotypes present in the reference sequence. Graph-based genomes, that integrate long read genome sequences for a subset of representative breeds and short read sequence data from hundreds of breeds and individuals to build a pangenome graph, provide an alternative, to capture global diversity. Graph-based pangenomes have recently been produced for other ruminants including, cattle (Crysnanto and Pausch 2020; Crysnanto et al. 2021; Talenti et al. 2022) and goats (Li et al. 2019), and a sheep pangenome graph which includes 13 breeds is also now available (Li et al. 2023). The graph-based pangenomes generated for cattle have been shown to increase read mapping rates, reduce allelic biases and identify structural variants with a high level of accuracy (Talenti et al. 2022). As such a graph-based genome for sheep incorporating many different breeds and populations spanning the depth and breadth of genetic diversity from across the globe, would provide a hugely informative research tool to inform future breeding and conservation strategies.

## Characterising global diversity in sheep populations using genomic resources

**Fig. 2:**
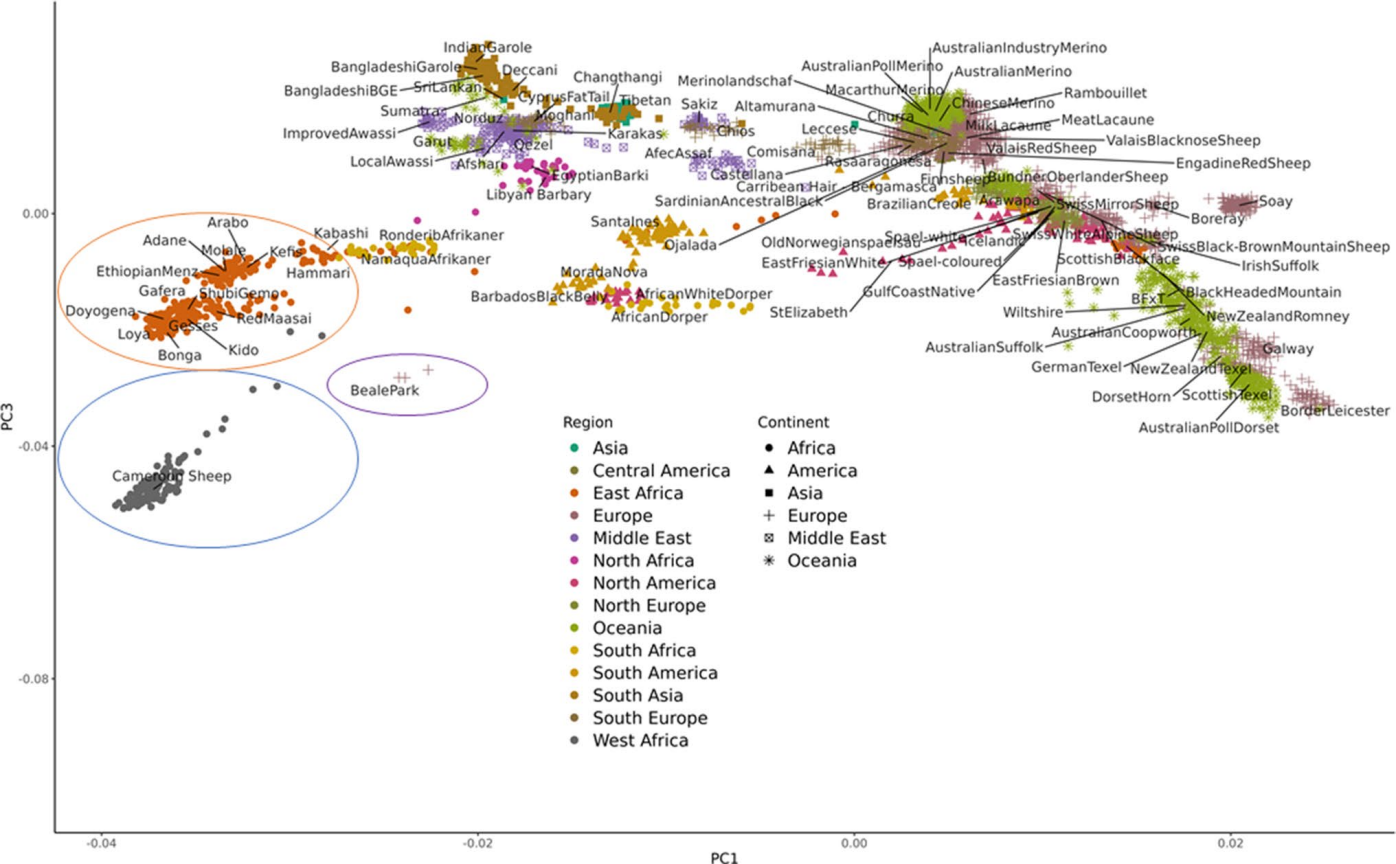
Principal component analysis illustrating the genetic diversity of sheep breeds from across the globe using 50K genotyping data (PC1 contributed 16% and PC3 7% to the variance). Included in the analysis are 50K genotypes from the HapMap dataset from Kijas et al. (2012), populations of East African sheep from Ahbara et al. (2019) (orange circle) and West and Central African sheep (blue circle) from Wiener et al. (2022). Cameroon sheep from a zoo collection at Beale Park (unpublished) are circled in purple

Before the development of long read sequencing and pangenomes, sheep benefitted from the availability of several genotyping tools, including the Illumina^®^ 50K Ovine Beadchip, both for the purposes of genomic selection, and for capturing genetic diversity using a set of genetic markers. The Illumina^®^ OvineSNP50 BeadChip was developed by the International Sheep Genomics Consortium (ISGC; www.sheephapmap.org; Kijas et al. 2009). Kijas et al. 2012 used the Illumina^®^ 50K chip to genotype 49,034 SNPs in 2819 animals from a diverse collection of 74 sheep breeds, generating the sheep HapMap dataset (https://www.sheephapmap.org/hapmap.php), which provided a global picture of the genetic history of sheep and variation across breeds. More recent studies have added 50K genotyping data from additional geographical locations and local and indigenous breeds not represented in the original HapMap dataset (Kijas et al. 2012), including from, Asia (Wei et al. 2015), Russia (Deniskova et al. 2018), India (Kumar et al. 2021) and Eastern Europe (Machová et al. 2023). Adding 50K genotypes from the African continent e.g. from North and East Africa (Ahbara et al. 2019) and West/Central Africa (Wiener et al. 2022), to the HapMap dataset, illustrates the unique diversity represented by these breeds and highlights the importance of including the diversity they represent in new genomic resources for sheep (Fig. 2). In addition, characterising the genetics of production breeds is also important to understand genetic relationships between breeds. The 50K chip has been used, for example, to characterise the genetic diversity of terminal sires in the US (Davenport et al. 2020) and the genetic diversity in New Zealand’s composite flocks has also been characterised using a higher density 600K chip (Brito et al. 2017b). When combined the genotyping datasets from SNP arrays for sheep now probably capture a considerable amount of the genetic diversity represented by sheep breeds from across the globe.

Genotyping data are also useful for conservation purposes. Many indigenous local breeds are now very rare, including for example, the Cameroon sheep from West/Central Africa. As such zoo populations often provide important reservoirs of genetic diversity that can be used for breed conservation (Woodruff 2001). The three ‘Beale Park’ individuals shown in Fig. 2 below are a trio of Cameroon sheep from a wildlife park collection in the UK. Although they are purportedly a “West/Central African” breed, these individuals originated from zoo populations that have been bred in Europe over several generations. Analysis of their 50K genotypes (shown in purple) reflect this, as they cluster some distance from the Cameroon sheep populations from West/Central Africa (shown in grey). As such their genetics may not be sufficiently representative of Cameroon sheep populations from West/Central Africa to be helpful for conservation purposes.

Analysing millions of variants from short read whole genome sequencing data can be even more informative. A wealth of short read whole genome sequencing data also now exists for sheep breeds and populations from across the globe. Li et al. (2020), for example, performed deep resequencing of 248 sheep, including wild *Ovis orientalis* landraces and improved breeds, and were able to detect genomic regions containing genetic variation of relevance to domestication, breeding, and selection. With additional whole genome sequencing data they were then able to define chromosomal evolution between wild, hybrid and domestic sheep (Li et al. 2022). Recently, Deng et al. 2020 also provided a comprehensive genomic analysis of haplotype diversity in the Y chromosome, mitochondrial DNA, and variants called from whole genome sequence data from 595 sheep representing 118 domestic populations.

Climate change and the pressures it will place on food production will shape future sheep populations and production systems, making characterising and conserving existing genomic diversity increasingly important (Georges et al. 2019). Short read whole genome sequencing data can provide a tool to investigate adaptation in populations of sheep living in diverse and extreme environments at the genomic level e.g. (Yang et al. 2016; Wiener et al. 2021). Wiener et al. 2021 identified over three million single nucleotide variants across twelve Ethiopian sheep populations and applied landscape genomics approaches to investigate the association between these variants and environmental variables. Yang et al. 2016 performed whole genome sequencing of 77 sheep living at varying altitudes and detected a novel set of candidate genes associated with hypoxia response at high altitudes and water reabsorption in arid environments. These studies illustrate how informative large-scale short read whole genome sequencing from diverse populations of sheep can be in identifying the genomic variation driving complex traits such as environmental adaptation and resilience in extreme environments. Harnessing the power of this functional variation will be important in future breeding strategies that aim to select for resilience traits to mitigate the effects of extremes of climate on sheep production systems.

The wealth of short read whole genome sequencing data for sheep provides a rich and diverse set of sequence information from which to call variants. There are several resources available to view and mine this data including iSheep: an integrated resource for sheep variant, phenotype and genome information (Wang et al. 2021). The Sheep Genomes Database (SheepGenomesDB) (https://sheepgenomesdb.org) houses the sequence variants called, using a standardised pipeline, from sheep short read whole genome sequencing data that has been deposited in the public archives. It is a hugely valuable community resource, not least because calling variants against the reference genome sequence takes a considerable amount of time and computational resource. Through the application of a single harmonised pipeline for read quality control, mapping, variant detection, and annotation, SheepGenomesDB makes available variant collections derived in a standardised manner against the reference genome. The recent change from the Texel Oar_v3.1 to the Rambouillet ARS-UI_Ramb_v2.0, as the community adopted reference genome sequence, has necessitated generating a new consensus set of variant calls for sheep. The third run of SheepGenomesDB will pull all the publicly available whole genome sequence data for sheep in the Short Read Archive (SRA) of sufficient depth and quality (from > 3000 animals) and call variants against ARS-UI_Ramb_v2.0. The new variant call set will be deposited in the European Variant Archive (EVA) with the other available variant call sets for sheep (https://ftp.ebi.ac.uk/pub/databases/eva/rs_releases/release_4/by_species/ovis_aries/). Once they are deposited in EVA variant tracks can be visualised against the available reference genomes, e.g. Rambouillet ARS-UI_Ramb_v2.0, using the Ensembl genome browser (Hunt et al. 2018). Generating this new set of consensus variant calls for sheep will provide a hugely useful set of genetic markers representing global genetic diversity.

Given the amount and diversity of short read whole genome sequencing data that is publicly available, it would now also be possible to generate a diverse haplotype reference panel for sheep, similar to those available for pig (Nosková et al. 2021) and cattle (Snelling et al. 2020), for imputation purposes. This resource would open-up a host of possibilities for low pass sequencing of many individuals capturing both between and within population diversity and providing the potential to improve genomic prediction by optimising the markers used in genomic evaluation.

## Genomic selection in sheep: integrating available genomic resources as a priori information in breeding programmes

A key component of improving profit and production output in sheep, particularly in Australia and New Zealand, has been the use of genomic selection (Daetwyler et al. 2010). Genomic selection is a form of marker-assisted selection in which genetic markers covering the whole genome are used to estimate an animal’s breeding value (Goddard and Hayes 2007). In sheep causative variants for production relevant traits with large phenotypic effects, have been successfully detected, using quantitative, population and molecular genetics approaches e.g. for carcass traits (Clop et al. 2006; Tellam et al. 2012; Matika et al. 2016). However, the majority of health, welfare and resilience traits, are polygenic and any causative variants are likely to have small effects, which makes detecting them more difficult (Georges et al. 2019). Functional genomic data can help enrich for variance in quantitative traits reviewed in (Johnsson 2023). Since most causal variants for complex traits are likely to be located in regulatory regions of the genome and will impact complex traits by changing gene expression (Tam et al. 2019) improvements in prediction accuracy could be achieved by filtering the genetic marker information, used for genomic selection, based upon whether the genetic variants reside in regulatory regions of the genome and then developing robust prediction models that can accommodate information about genome function (Georges et al. 2019).

Recently, new methods for integrating genomic information, such as gene expression or methylation data, into genomic prediction models have been proposed e.g. (Xiang et al. 2019, 2021). These multi-layered models, which are based on the combination and ranking of many types of functional genomic data from multiple individuals, have been shown for cattle to facilitate further improvements in predicting genetic merit and consequently on genomic selection (Xiang et al. 2019, 2021). Liu et al. 2022 also recently demonstrated the feasibility of linking variants associated with complex traits from GWAS with gene expression and regulation information across tissues and cell types in cattle, for the cattle GTEx project. The FarmGTEx project (https://www.farmgtex.org/) has now extended these efforts to pig (The FarmGTEx-PigGTEx Consortium 2023) and chicken (Pan et al. 2023) and plan a similar initiative for sheep. A priority for the sheep genomics community going forwards will be generating suitable datasets for this purpose. There are currently only a handful of datasets for sheep with matched genotypes and RNA-Seq data for tissues, that can be used to train the models for FarmGTEx, such as a recently published expression QTL study from muscle and liver for carcass traits (Yuan et al. 2021b). The opportunity does now exist, however, to generate gene expression information at a population scale due to a reduction in cost of RNA-sequencing and the development of new assays that are deployable at scale such as Illumina 3′-sequencing. The challenge for sheep may also be accessing phenotype data for trait prediction as recording in sheep is much less advanced across traits than for cattle, pig, and chicken. However, accurate recording to inform selection strategies will become increasingly important as future extremes of climate put pressure on producers to select animals that are more resilient.

## New genomic resources can inform genome editing and the use of sheep as biomedical models

While genomic selection is likely to provide the foundation of many future commercial breeding programmes for sheep, it is limited by the genetic pool of the population under selection. If a target trait is not encoded in the genome of a breeding population, then it is not possible to select for it. Genome editing has the potential to offer an effective solution to this problem (McFarlane et al. 2019). Sheep are particularly amenable to genome editing and it has been applied successfully for a small number of production relevant target genes, reviewed in Proudfoot et al. (2015). Advances in the genomic resources for sheep will provide information to identify new editing targets particularly those that control breed-specific characteristics that may be present in one breeding population but not in another. One example is the ‘polled’ or hornlessness trait that is a distinct characteristic of some breeds such as the Poll Dorset. Horns can cause injury both to the sheep themselves and to their handlers and consequently, particularly in production animals, polledness is desirable. However, some production breeds with desirable resilience and sustainability traits, like the Wiltshire Horn, a wool-shedding breed with a good carcass and high feed efficiency, have undesirable large horns that make them difficult to handle and manage. Gene editing for polledness has been achieved successfully in cattle, reviewed in (Van Eenennaam 2019), but in sheep is likely to be more complex, reviewed in Simon et al. (2022). A 1.78Kb insertion in the 3′ UTR region of the *RXFP2* gene on chromosome 10 has been identified which is strongly associated with polledness in GWAS (Wiedemar and Drögemüller 2015) however it does not segregate in the same way across all breeds (Lühken et al. 2016). Comparative approaches to analyse breed-specific genomic resources for sheep, across individuals and populations, will help to reveal the functional basis of traits present in one breed or population that are desirable in another providing novel targets for selective breeding and/or genome editing (Clark 2022).

In addition to their role as food production animals sheep are also important biomedical models (Banstola and Reynolds 2022). The new highly contiguous ARS-UI_Ramb_v2.0 reference genome and associated annotation, provides a research tool that can inform studies designed to identify alleles encoding human physiological processes and diseases. One recent example, is the novel sheep model of *CLN1* disease, in which gene editing was used to insert a disease-causing *PPT1 (R151X)* human mutation into the orthologous sheep locus (Eaton et al. 2019; Nelvagal et al. 2022). New tools including high-throughput CRISPR/Cas9 knock-out libraries, such as those available for pigs e.g. (Yu et al. 2022), will help considerably with identifying novel alleles for genome editing in both human and farmed animal studies. At present, however, a lack of suitable primary cell lines for sheep is a barrier to progress. As the applications of genome editing technologies in the biomedical field expand a high quality annotated reference genome for sheep on which to base target selection will become even more useful.

## The future

In addition to the new genomic resources for sheep described above there are further exciting developments on the horizon (Fig. 1). For example, recent improvements in tools and resources for long read sequencing have made assembling fully contiguous assembled telomere-to-telomere genomes possible. The human telomere-to-telomere genome assembly is a revolutionary new tool for human research unlocking the complex regions of the genome to study genome function and genetic variation (Nurk et al. 2022). A telomere-to-telomere reference genome assembly for sheep is currently being generated for the Ruminant Telomere-to-Telomere project which is led by the USDA and University of Idaho.

From a transcriptome perspective, since publication of the sheep gene expression atlas, expanded transcriptomes, that include histological tissue maps and characterisation of all RNA populations, have been published, e.g. for pig (Jin et al. 2021), and similar new resources of this type for sheep will soon follow. Furthermore, long read RNA isoform sequencing technologies, can now capture full-length isoform information, even at single cell level resolution. These technologies make transcript annotation considerably easier and allow for the characterisation of splicing events and prediction of full-length open reading frames. Isoform sequencing (Iso-Seq) data for a small subset of tissues is available for sheep, for the purposes of annotating the Rambouillet genome, and from a small number of published studies that have focussed on specific tissues relevant to phenotypes of interest (Yuan et al. 2021a, 2022). New long read isoform sequencing datasets for multiple tissues, cell types and developmental stages, will provide a valuable novel resource for genome annotation and build on the transcriptomic resources already provided by short read RNA-Seq data. Long read sequencing technologies will also facilitate, the generation of breed- specific transcriptomes. These breed-specific transcriptomes based on full-length isoform information, will allow the classification of sets of pan-genes and pan-transcriptomes for sheep providing new insights into how isoform usage can influence key traits across different breeds.

The primary challenge facing the sheep and wider farmed animal genomics community now is harnessing the power of a highly accurate reference genome with functional genomics data, at a population scale, and from there how to leverage this information to enhance genomic prediction reviewed in (Johnsson 2023). The potential to go ‘beyond the genome’ by using epigenetic modifications to predict genetic merit also shows significant potential, reviewed in (Clarke et al. 2021). DNA methylation arrays, for example, have proved to be useful tools for informing breeding programmes for sheep, and provide an opportunity to accelerate the physiological response of breeding populations to environmental pressures (Clarke et al. 2021). Tools to visualise the combination of genetic variation with predicted function will be critical in advancing the sheep genomics field. Functional genomic comparisons of different sheep breeds will become increasingly powerful as haplotype-resolved reference genomes and pangenomes with matched functional annotation data become the new standard for sheep and other farmed animal species.

## Conclusions

The field of sheep genomics has undoubtedly moved into a new era. New functional annotation datasets for sheep for many different tissues and cell types provide new resources to link cell, tissue and whole animal scale knowledge. Novel opportunities also now exist for interrogating gene regulation information at single cell resolution providing a much more complete picture of transcriptional complexity in sheep. Affordable long read sequencing technologies have caused an explosion in the number of new genome assemblies that are being generated for many different breeds and populations. Genetic improvement in the future will also almost certainly include the use of pangenomes to understand and visualise the diversity of farmed animal genomes (Hayes and Daetwyler 2019). For this reason, pangenome efforts should ensure they capture the global genetic diversity of sheep breeds, including those from the global south. Logistical considerations will inevitably arise with the rapid expansion of genomes and genomic resources for sheep. Genome browsers, such as Ensembl, will need to keep pace with how rapidly these new genomic and transcriptomic resources are being generated. This will need to happen quickly in order that the community can maximise the benefit of this new information, and will require resources, effort and funding (Cunningham et al. 2022). The sheep genomics research community will also need to work with stakeholders to decide what the priorities are for the coming decade. These priorities should be centred around providing resources that can inform global sheep breeding systems in a way that will help to accelerate their response to future extremes of climate, produce healthier improved animals and provide enough food for a growing human population.
